# Immune sensitization of equine bronchus: glutathione, IL-1β expression and tissue responsiveness

**DOI:** 10.1186/1465-9921-6-104

**Published:** 2005-09-15

**Authors:** MG Matera, L Calzetta, A Peli, A Scagliarini, C Matera, M Cazzola

**Affiliations:** 1 Department of Experimental Medicine, Unit of Pharmacology, 2^nd ^University of Naples, Naples, Italy; 2 Department of Respiratory Medicine, Unit of Pneumology and Allergology, A. Cardarelli Hospital, Naples, Italy; 3 Department of Veterinary Clinical Science, Faculty of Veterinary Medicine, University of Bologna, Bologna, Italy; 4 Department of Veterinary Public Health and Animal Pathology, Faculty of Veterinary Medicine, University of Bologna, Bologna, Italy; 5 Department of Pharmaceutical Sciences, Santissima Annunziata Hospital, Chieti, Italy

**Keywords:** equine bronchi, passive sensitization, IL-1β, reduced-glutathione

## Abstract

**Background:**

Increasing clinical epidemiological and experimental evidence indicates that excess of production of reactive oxygen free radicals (ROS) induced by an oxidative stress is involved in the pathogenesis of a number of human airway disorders, as well as equine recurrent airway obstruction. Free-radicals modulate the activation of transcription factors, such as nuclear factor-(NF)-κB and activator protein (AP)-1, in several different cells. This activation leads to expression of many pro-inflammatory cytokines, including interleukin (IL)-1β. We have hypothesized that equine airway sensitization might induce an oxidative stress and increase the ROS production, which in turn might enhance a production of IL-1β and airway hyperresponsiveness.

**Methods:**

We have examined the effect of passive sensitization on IL-1β mRNA expression and electrical field stimulation (EFS)-induced contraction in equine isolated bronchi, and the potential interference of reduced-glutathione (GSH), an antioxidant, with these responses. Bronchi passively sensitized with serum from animals suffering from heaves and having high total level of IgE, and control tissues, either pretreated or not with GSH (100 μM), were used to quantify IL-1β mRNA. Other tissues were used to study the effect of EFS (3–10–25 Hz).

**Results:**

Mean IL-1β mRNA expression was higher in passively sensitized than in control rings. GSH significantly (*p *< 0.05) reduced the IL-1β mRNA expression only in passively sensitized bronchi. ELF induced a frequency-dependent contraction in both non-sensitized and passively sensitized tissues, with a significantly greater response always observed in sensitized tissues. GSH did not modify the EFS-induced contraction in non-sensitized bronchi, but significantly (*p *< 0.05) decreased it in passively sensitized tissues.

**Conclusion:**

Our data indicate that the passive sensitization of equine bronchi induces inflammation and hyperresponsiveness. These effects might be due to an oxidative stress because a pretreatment with GSH decreased the increased IL-1β mRNA expression and responsiveness to EFS of passively sensitized bronchi.

## Introduction

Increasing clinical epidemiological and experimental evidence indicates that excess of production of reactive oxygen free radicals (ROS) is involved in the pathogenesis of a number of airway disorders [1]. When airway cells and tissues are exposed to oxidative stress elicited by environmental pollutants, infections, antigen challenge, inflammatory reactions or decreased level of antioxidants, the enhanced production of ROS can negatively affect several chronic obstructive airway diseases, including human asthma and COPD and equine recurrent airway obstruction (RAO) [2-4]. Horses suffering from RAO have a decreased pulmonary antioxidant capacity, which may render them more susceptible to oxidative challenge [5].

The importance of ROS in inducing RAO is documented by the fact that isoprostanes, which are markers of lipid peroxidation [7], are increased by pulmonary oxidative stress induced by strenuous exercise in heaves-horses and, moreover, epiPGF_2α _is significantly augmented in these horses during an exacerbation and correlated with airway inflammation and pulmonary function [8,9]. It must be highlighted that experimental airway antigen challenge is associated with immediate formation of ROS, which persists throughout the late asthmatic response [10] and, at least in rats, antigen exposure also increases lipid peroxidation in bronchoalveolar lavage [6].

This resulting oxidative stress may lead to the induction of redox-sensitive transcription factors such as nuclear factor (NF)-κB, hypoxia inducible factor (HIF) and activator protein (AP)-1 in several different cells that leads to an increased expression of pro-inflammatory cytokines, including interleukin (IL)-1β [11,12]. Interestingly, IL-1β plays an autocrine role in altered responsiveness of atopic asthmatic sensitized airway smooth muscle, at least in humans [13].

High levels of NF-κB activity are found during crisis in heaves-susceptible horses [14,15]. Therefore, it is not surprising that exacerbation of the disease in heaves-susceptible horses coincides with increased mRNA expression in BAL cells of the pro-inflammatory cytokines IL-1β and tumor necrosis factor (TNF)-α [16].

Airways that have been passively sensitized with serum from atopic asthmatic patients can be used to study pathophysiological mechanisms that underlie the induction of airway inflammation and hyperresponsiveness [17,18].

We hypothesized that airway sensitization might induce an oxidative stress and increase the ROS production, which in turn might enhance a production of IL-1β and airway hyperresponsiveness. Therefore, this study aimed to evaluate the effect of passive sensitization on IL-1β mRNA expression and on contraction induced by electrical field stimulation (EFS) in equine isolated bronchi and to explore the effect of an antioxidant, the reduced-glutathione (GSH), on these responses.

## Materials and methods

### Tissue preparations

Four healthy equine male lungs (aged 1.7 ± 0.09 years; weighted kg 390 ± 64.9) were obtained from local abattoir; all animals showed a negative history of heaves. Immediately, after resection, 3^rd ^generation bronchi were excised, cleaned and cut in rings.

### Passive sensitization

Tissues were passively sensitized in a random manner, as described by Schmidt et al. [19]. Briefly, equine bronchial smooth muscle rings were rotated overnight at room temperature in tubes containing modified Krebs Henseleit buffer (KH; composition in mM. 118.4 NaCl, 25.0 NaHCO_3_, 11.7 dextrose, 4.7 KCl, 2.6 CaCl_2_, 2H2O, 1.19 MgSO_4_·7H_2_O and 1.16 KH_2_PO_4 _with a cyclo-oxygenase inhibitor; indomethacin; 5 μM; pH 7,4,) in the absence (control rings) or presence of sensitizing serum (sensitized rings). The serum was prepared from the whole blood of animals suffering from heaves, during an exacerbation, with a total level of IgE of 8,095.04 ± 90.9, measured as described by Tizard [20]. Sera were not pooled but were frozen at -20°C in 200-ml aliquots until required.

In order to evaluate the role of ROS in sensitized airways, some of control tissues or sensitized ones were treated with GSH (100 μM). The following morning rings were transferred to 10 ml organ baths aerated with 5% CO_2 _and 95 O_2_%, at 37°C; containing modified KH buffer, which was changed every 10 min. The isometric changes in tension were measured with a transducer Fort 10 WPI (Basile, Instruments, Italy)

### Lymphocyte proliferation assay

Lymphocyte proliferation assay was carried out in duplicate wells of a 6 well flat bottomed plates (Costar, MA) using horse peripheral blood mononucler cells (PBMCs). For PBMC isolation, blood samples (10 ml) were collected from a healthy horse; cells were then isolated using the standard Ficoll-Hypaque method, and washed three times in PBS. PBMCs were seeded by centrifugation and suspended in RPMI 1640 medium (GIBCO) supplemented by 10% inactivated SFB, and antibiotics (penicillin/ml 200 IU and 150 μg/ml streptomycin) at a final concentration of 2 × 106 cells/ml. For lymphocyte stimulation assays (LSAs), 1 ml of cell suspension along with either 1 ml of ConA (extract from *Concanavalia ensiformis*, ICN Biochemicals, Cleveland, Ohio, USA) at 10 μg/ml or 1 ml of medium alone were added to 6 well plates The plates were incubated at 37°C and 5% CO2 for 18 or 24 hours. Duplicate cultures were set up to separately measure cytokine production and cellular proliferation. Total RNA was isolated from either stimulated or non stimulated PBMCs cultures using the RNeasy tissue kit (Qiagen Inc., Valencia, CA, USA). RNA concentration was measured by optical density. RNA samples were treated with amplification grade DNAse I (Amersham) to remove any traces of genomic DNA. RT PCR was performed in order to amplify IL1β mRNA using the specific primers FIL-1β 5'GAGGCAGCCATGGCAGCAGTA3' and RIL-1β 5'TGTGAGCAGGGAACGGGTATCTT3' that were designed on the basis of the horse IL1β sequence published in the GENBANK database (D42147). The RT PCR was performed using the Reverse iTtm one step RT-PCR kit (ABgene), briefly, RNA of each sample was reverse transcribed at 47°C for 30' and then incubated at 95°C for 5'. The resulting cDNA was amplified in 35 cycles of denaturation at 94°C for 30 sec, annealing at 58°C for 30 sec and extension at 72°C for 30 sec, followed by a final extension step at 72°C for 7 min. The amplicons were subsequently analyzed on a 1% agarose gel stained with ethidium bromide using Fluor S Multimager (Biorad USA).

### Standard DNA preparation

The specific fragment of 257 bp of horse IL1β gene previously amplified from ConA stimulated PBMCs was cloned into the pCR 4/TOPO vector using TOPO cloning kit (Invitrogen) and purified with Turbo Kit (QBIOgene). The recombinant plasmid was linearized upstream the target sequence using the restriction endonuclease PmeI (Fermentas) to avoid the presence of supercoiled plasmid and to simulate more closely the amplification efficiency of genomic DNA. The linearized plasmid was visualized and quantified by electrophoresis in 1% agarose gel containing ethidium bromide using 2-log DNA ladder (New England BioLabs). 10-fold dilutions of the plasmid were made representing 1,14 × 109 copies of DNA/μl of template and stored at -80°C until required. The PCR standard curve was constructed by plotting the plasmid DNA dilutions against the corresponding QPCR threshold cycle value. The threshold was determined using the Auto-Find Threshold function of the Rotor-Gene 3000 (Corbett Research, AU) that scans the range of threshold levels to obtain the best fit of the standard curve through the samples that have been defined as standards.

### Expression of mRNA for IL-1β in equine isolated bronchi

RNA was extracted from all mucosal specimens by using RNeasy tissue kit (QIAgen Inc., Valencia, CA, USA). The total RNA was isolated and quantified by spectrophotometry and the ratio 260/280 was estimated. The purified mRNA was incubated for 30 minutes at 37°C with DNAse I (Amersham Biosciences) to eliminate genomic DNA. 50 μg of mRNA was reverse transcribed at 37°C for 60' using the Sensicript reverse transcriptase (Qiagen) and specific reverse primer RIL-1β'. The resulting cDNA was then amplified by real time PCR. The quantitative PCR assay was developed to allow an absolute quantification of the cDNA. The reaction was carried out on the Rotor-Gene 3000 system (Corbett Research, Australia), using QuantiTect SYBR Green PCR kit (Qiagen). Each sample was amplified in a final volume of 20 μl containing 10 μl of master mix, 20 pmol of primers FIL1β and RIL1β and 5 μl of cDNA. Cycling parameters were 15 min at 95°C for the hot start TAQ polymerase activation followed by 45 cycles of 15 s at 94°C, 20 s at 58°C and 20 s at 72°C. Fluorescence data acquisition was carried out at the end of each PCR extension phase. The reaction was performed testing the samples and 5 standard plasmid dilution in duplicate.

### Electrical field stimulation studies (EFS)

Tissues were allowed to equilibrate passively for 90 min. During equilibration (2 h) optimal passive tension was determined by gentle stretching of tissue (≅2 mg). The tissue responsiveness was assessed by acetylcholine (ACh) 100 μM; when the response reached a plateau, rings were washed tree times and allowed to equilibrate for 30 min. After full recovery of the tissues, EFS was performed by placing tissues between two wire platinum electrodes, connected to a stimulator 3165 multiplexing pulse booster (Basile Instruments, Italy). Tissues were electrically stimulated for 10 sec (10 V, 0.2 ms) by increasing EFS frequencies (3–10–25 Hz). At the end of the experiments, the wet weight of each tissue was determined.

### Analysis of results

Contractile response are expressed as percentage of ACh (100 μM) induced contraction. All values are presented as mean ± SEM. All n values refer to the number of the lungs. Statistical significance was assessed by multifactorial analysis of variance (ANOVA). In presence of a significant overall ANOVA, whenever there was significance, the Tukey's Multiple Comparision test was applied at the 5% level for comparison of the means. A probably level of *P *< 0.05 was considered as significant for all tests. All data analysis was performed using computer software (GraphPad Prism, CA, USA).

### Drugs

The following drugs were used: acetylcholine (ACh), indomethacin and GSH (Sigma, Chem. Co. St Louis, MO).

## Results

### Baseline characteristics of the bronchial rings

There was no significant difference (*P *> 0.05) between passively sensitized and non-sensitized bronchi in absence or in presence of a treatment with GSH (100 μM) in wet weight or contraction induced by ACh 100 μM (table 1).

**Table 1:** Wet weight and contraction induced by ACh (100 μM.) in equine non-sensitized (C) and passively sensitized isolated bronchi, in absence or in presence of a treatment with reduced-glutathione (G; 100 μM). All values are mean ± SEM of four samples

	**C**	**C+G**	**S**	**S+G**
**Wet weight mg**	285 ± 42.2	295 ± 39	285 ± 30.1	254 ± 27.6
**ACh contraction (gr)**	15.80 ± 3.4	13.70 ± 0.4	16.40 ± 0.9	13.20 ± 0.5

### Effect of passive sensitization and GSH on expression on mRNA for IL-1β

**Figure 1:**
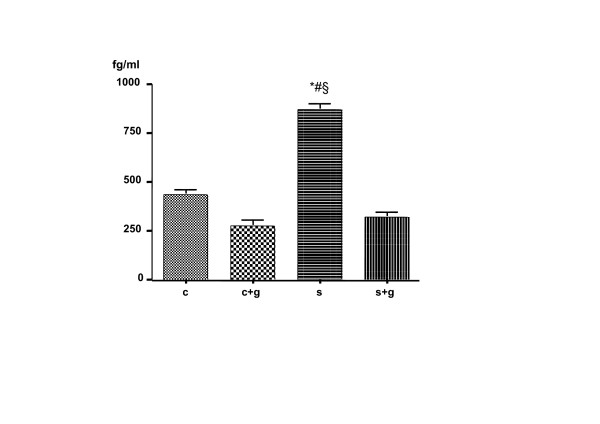
Effects of passive sensitization and reduced-glutathione (G; 100 μM) on IL-1β mRNA. Values are mean ± SEM of four samples. C: control rings; S: passively sensitized rings. *P < 0.05 C vs. S; #*P *< 0.05 C+G vs. S; §*P *< 0.05 S+G vs. S.

**Figure 2:**
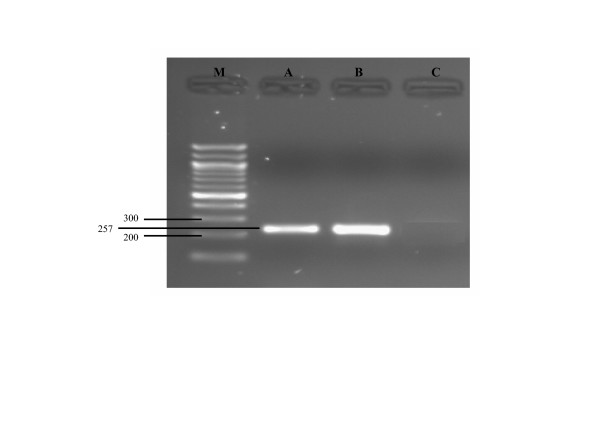
Electrophoresis of IL1b RT-PCR product. Specific 257 bp fragments amplified from PBMCs after 18 and 24 hours stimulation with ConA (lane A and B), lane C no template control, lane M molecular weight marker 100 bp

Mean IL-1β mRNA expression in equine passively sensitized bronchi was higher when compared to the control rings. A pretreatment with GSH did not modify the IL-1β mRNA expression in non-sensitized bronchi, whereas it significantly reduced the IL-β mRNA expression in passively sensitized ones (Figure 1 and 2).

### Effect of passive sensitization and GSH on EFS responses

**Figure 3:**
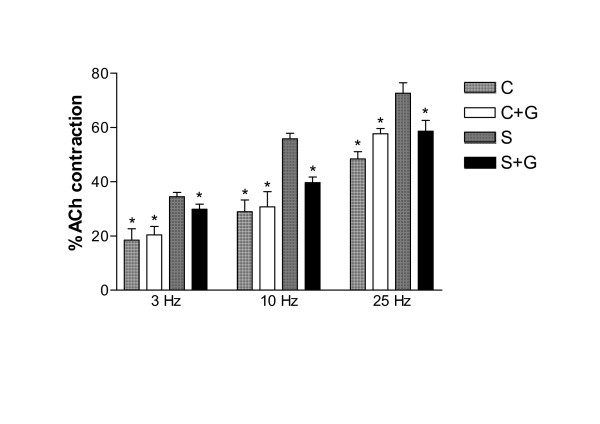
Effects of passive sensitization and reduced-glutathione (G; 100 μM) on contraction induced by electrical field stimulation in equine isolated bronchi. Values are expressed as percentage of ACh (100 μM) induced contraction and are mean ± SEM of four samples. C: control rings; S: passively sensitized rings. *P *< 0.05 vs. S.

EFS induced a contraction, frequency dependent, in both non-sensitized and passively sensitized tissues. The magnitude of this contraction was significantly greater in serum-sensitized tissues than in non-sensitized ones at each frequency used. (figure 3). A pretreatment with GSH did not modify the EFS induced contraction in non-sensitized bronchi (figure 3). In passively sensitized equine bronchial rings, a pretreatment with GSH (100 μM) significantly (*P *< 0.05) decreased contraction at all frequencies used compared to passively sensitized tissues (figure 3).

## Discussion

It has been demonstrated that passive sensitization of isolated airway smooth muscles increases the contractile responses to several agonists, such as histamine and leukotriene C_4 _[19]. This effect is independent of specific IgE and appears to be related to some other factors associated with serum containing high concentrations of total IgE [17,19,21].

The increased reactivity of airways could be due to an increased production of ROS, a term that includes various oxygen free radicals (superoxide anions, hydroxyl radicals, hydrogen peroxide, hypochlorous acid, peroxynitrite and ozone) released from alveolar macrophages, neutrophils and eosinophils when exposed to oxidative stress elicited by inflammatory reactions, infections, environmental pollutants or antigen challenge [22]. In effect, studies carried out in asthmatic patients have demonstrated that antigen challenge enhances the production of ROS. Leukocytes obtained from asthmatic patients generate more ROS compared to cells derived from control subjects [23]. Also neutrophils and monocytes purified from blood of asthmatic patients possess a higher propensity to release ROS than cells obtained from controls [24]. Oxidative stress induced by IgE challenge leads to the activation of genes for many pro-inflammatory cytokines, including IL-1β, which plays an important role in airway hyperresponsiveness and this not only in man but likely even in heaves [2,12,16,25-27].

In the present study, equine passively sensitized isolated bronchi showed a greater expression of IL-1β mRNA than non sensitized tissues. Our result fits with the *in vitro *documentation that oxidants cause the release of inflammatory mediators such as TNF-α, IL-8 and IL-1-β [26,27]. This is a phenomen that has also been documented in horses. In fact, Giguere et al. [16] demonstrated that the IL-1β mRNA expression was significantly higher after exposure to moldy hay in horses with heaves when compared to values obtained during clinical remission or in healthy controls in one out of two trials.

The sensitized bronchial rings revealed also an increase in the contractile response induced by EFS, which might be related to an altered neural functioning. In human isolated airways, Ichinose et al. [28] demonstrated that the incubation of bronchi with IgE enhanced cholinergic neurotransmission via a M_2 _dysfunction, without affecting the responses to exogenously applied ACh. This finding is likely related to an indirect mechanism involving inflammatory cell-derived mediators, such as cytokines, and ROS, which can indirectly influence contractile responses related to an alteration of airway nerve function. An increased cholinergic nerve-induced contraction induced by chemical oxidants has been documented in rat tracheal smooth muscle too [29]. On the other hand, ROS also elicit a direct effect on airway smooth muscles, acting either as contractant or relaxant agents. *In vitro*, it has been documented that exposure of guinea pig tracheal tissues to peroxynitrite and hypochlorous acid causes hyperresponsiveness to histamine and substance P [30,31] and that the oxidative stress increases the contraction of isolated human airway smooth muscles [32]. In agreement with these findings, our results demonstrated that passive sensitization enhances cholinergic nerve-induced contraction, without affecting contractile responses induced by exogenous ACh in equine bronchial rings. In contrast, they did not confirm the previous documentation of Olszewwski et al [33] that in equine trachea, hydrogen peroxide reduced exogenous spasmogen-induced contraction. This discrepancy can partially be explained by the fact that in the study of Olszewski et al [33] only single rather synergistic action of free-radicals in the trachea was examined, whereas our findings have been obtained on isolated bronchi.

In order to confirm the hypothesis that airways inflammation and hyperresponsiveness observed in equine passively sensitized bronchi could be related to an increased production of ROS, we evaluated the effects of a pretreatment with GSH on either non-sensitized or passively sensitized bronchi. We observed that a pretreatment with GSH significantly decreased the IL-1β mRNA expression and the contraction induced in passively sensitized tissues, but did not show any effect in the non-sensitized ones. In order to justify these different responses, we must consider that under normal circumstances ROS are kept under tight control by SOD enzymes [26]. In acute and chronic inflammation, the production of ROS increases at a rate that overwhelms the capacity of the endogenous defense system to remove them [26]. Moreover, GSH is an important component of the lung antioxidant defense [27]. Therefore, a supplementation of this antioxidant agent is necessary to protect lungs from oxidative stress during acute or chronic inflammation. Anyway, there is evidence that also other antioxidant agents, such as N-acetyl-cysteine (NAC), block both *in vitro *and *in vivo *the release of inflammatory mediators from epithelial cells and macrophages by a mechanism involving intracellular GSH and decreasing NF-κB activation [34].

In conclusion, our results indicate that passive sensitization of equine bronchi induces airway inflammation and hyperresponsiveness. These effects might be due to an oxidative stress. In fact, the increased IL-1β mRNA expression and responsiveness to EFS of passively sensitized bronchi were decreased by a pretreatment with GSH, which is an antioxidant agent. It is not a surprise that the increase in IL-1β mRNA expression was linked to an increase in airway hyperresponsiveness. In fact, it has been documented that pre-incubation of human isolated small bronchi with IL-1β increased responsiveness to substance P [35] and, moreover, this cytokine induced hyperresponsiveness in sensitized Brown-Norway rats [36]. However, from our study any proper relationship between the increase in IL-1β mRNA expression and the in smooth muscle contractility cannot clearly been seen and we cannot denied that GSH inhibited both in an independent manner. Therefore, further studies will better explain this finding.
